# Identification of an EMT-Related Gene Signature for Predicting Overall Survival in Gastric Cancer

**DOI:** 10.3389/fgene.2021.661306

**Published:** 2021-06-24

**Authors:** Weiyu Dai, Yizhi Xiao, Weimei Tang, Jiaying Li, Linjie Hong, Jieming Zhang, Miaomiao Pei, Jianjiao Lin, Side Liu, Xiaosheng Wu, Li Xiang, Jide Wang

**Affiliations:** ^1^Guangdong Provincial Key Laboratory of Gastroenterology, Department of Gastroenterology, Nanfang Hospital, Southern Medical University, Guangzhou, China; ^2^Department of Gastroenterology, Longgang District People’s Hospital, Shenzhen, China

**Keywords:** EMT, gastric cancer, LASSO, prognosis, signature

## Abstract

**Background:**

It has been widely reported that epithelial-mesenchymal transition (EMT) is associated with malignant progression in gastric cancer (GC). Integration of the molecules related to EMT for predicting overall survival (OS) is meaningful for understanding the role of EMT in GC. Here, we aimed to establish an EMT-related gene signature in GC.

**Methods:**

Transcriptional profiles and clinical data of GC were downloaded from The Cancer Genome Atlas (TCGA). We constructed EMT-related gene signature for predicting OS by using univariate Cox regression and least absolute shrinkage and selection operator (LASSO) regression analyses. Time-dependent receiver operating characteristic (ROC), Kaplan-Meier analysis were performed to assess its predictive value. A nomogram combining the prognostic signature with clinical characteristics for OS prediction was established. And its predictive power was estimated by concordance index (C-index), time-dependent ROC curve, calibration curve and decision curve analysis (DCA). GSE62254 dataset from Gene Expression Omnibus (GEO) was used for external validation. Quantitative real-time PCR (qRT-PCR) was used to detected the mRNA expression of the five EMT-related genes in human normal gastric mucosal and GC cell lines. To further understand the potential mechanisms of the signature, Gene Set Enrichment Analysis (GSEA), pathway enrichment analysis, predictions of transcription factors (TFs)/miRNAs were performed.

**Results:**

A novel EMT-related gene signature (including ITGAV, DAB2, SERPINE1, MATN3, PLOD2) was constructed for OS prediction of GC. With external validation, ROC curves indicated the signature’s good performance. Patients stratified into high- and low-risk groups based on the signature yielded significantly different prognosis. Univariate and multivariate Cox regression suggested that the signature was an independent prognostic variable. Nomogram for prognostication including the signature presented better predictive accuracy and clinical usefulness than the similar model without risk score to some extent with external validation. The qRT-PCR assays suggested that high expression of the five EMT-related genes could be found in human GC cell lines compared with normal gastric mucosal cell line. GSEA and pathway enrichment analysis revealed that focal adhesion and ECM-receptor interaction might be the two important pathways to the signature.

**Conclusion:**

Our EMT-related gene signature may have practical application as an independent prognostic factor in GC.

## Introduction

Gastric cancer (GC) is the third leading cause of cancer-related death and the fifth most common malignancy worldwide, with over 1,000,000 new patients and an estimated 783,000 deaths in 2018 (Bray et al., 2018). 39% of GC patients were identified with metastatic diseases (Thomassen et al., 2014). Patients with metastasis tended to have poor survival (Sleeman and Steeg, 2010). The increased chemotherapy in patients with metastasis didn’t increase the population-based overall survival (OS) (Bernards et al., 2013). To date, the TNM stage system is widely regarded as a guideline for survival estimate. But wide variation in prognosis exists among GC patients with the same TNM stage on account of the inherent heterogeneity (Jiang et al., 2017, 2018). Hence, novel strategies are needed to improve the survival prediction and further guide individual treatment in GC.

Epithelial-mesenchymal transition (EMT) is a reversible process in which epithelial cells can transdifferentiate into motile mesenchymal cells, and it is vital to embryogenesis, wound healing and the tumorigenic process (Dongre and Weinberg, 2019). EMT is a complicated process which can be driven by key transcriptional factors like SNAIL, zinc-finger E-box-binding (ZEB) and basic helix-loop-helix (bHLH) transcriptional factors (Peinado et al., 2007). And reprogramming of gene expression, lots of pathways such as transforming growth factor-β (TGF-β) family signaling, PI3K-AKT, ERK-MAPK, p38 MAPK and JUN N-terminal kinase (JNK) pathways, etc., are involved in EMT (Lamouille et al., 2014). EMT is associated with stemness, initiation, invasion, metastasis and chemo-resistance in GC, and the status of EMT is a critical prognosticator for GC (Murai et al., 2014; Huang L. et al., 2015). Due to the convenient access to transcriptional data from online data hubs, establishing the gene signature underlying the mechanism of cancer is an area of active research (Wang et al., 2019b; Zhao et al., 2019; Cao et al., 2020). Considering that EMT status has been previously shown to be prognostic in GC, biomarkers related to EMT represent a promising source for assembling an independently significant prognostic signature for GC.

In this study, we constructed an EMT-related gene signature for predicting OS based on the transcriptional profiles of GC from The Cancer Genome Atlas (TCGA). Univariate Cox regression and least absolute shrinkage and selection operator (LASSO) regression were conducted to identify the prognostic five-gene signature. Receiver operating characteristic (ROC) curve and survival analysis were used to estimate it. Then, a nomogram was built by combining the risk score and clinical parameters to predict OS in GC. Concordance index (C-index), ROC curve, calibration curve and decision curve analysis (DCA) were performed to assess the nomogram. Besides, the prognostic value of the nomogram was verified by an external validation. Collectively, our finding highlights the functional role of EMT-related gene signature and nomogram in predicting OS for GC.

## Materials and Methods

### Data Collection

RNA-sequencing and clinical information of GC samples in TCGA were obtained from the Genomic Data Commons Data Portal (GDC^1^). “HTSeq-FPKM” workflow type of transcriptome profiling for TCGA-STAD (stomach adenocarcinoma) project was download, including 375 cancer tissues samples’ and 32 normal samples’ gene expression profiles. Clinical information of 443 GC tissues from TCGA-STAD project was downloaded with the format of “bcr xml.” The TCGA-STAD cohort was assigned as the training cohort. The external validation cohort GSE62254 was acquired from Gene Expression Omnibus (GEO^2^) (Cristescu et al., 2015). GSE62254 was conducted by GPL570 platform (Affymetrix Human Genome U133 Plus 2.0 Array), consisting of 300 GC samples with corresponding clinical information. The normalized expression matrix GSE62254 was used directly for subsequent analyses. All the data was obtained in March 2020. Patients who met the following criteria were included in the subsequent analyses: (a) sufficient gene expression information, (b) survival time no less than 30 days, (c) sufficient clinical information of age, gender, TNM stage, T stage, N stage, M stage, number of lymph nodes examined and number of positive nodes. Increasing evidences have revealed that lymph node ratio (LNR, the ratio of the positive lymph nodes positive to lymph nodes examined) was an important prognostic factor in GC, so it was considered in our study and the LNR values of all patients included were calculated (Zhao et al., 2016; Lee et al., 2017). Thus, 278 patients (278 tumor samples and 26 normal samples) in TCGA-STAD and 298 patients (298 tumor samples) in GSE62254 were included in our study with the accompanying information above. For our study was based on the de-identified data from the TCGA and GEO databases, institutional review, institutional approval and informed consent were not required.

### Identification of Differentially Expression EMT-Related Genes (DEEGs)

Gene members from gene set HALLMARK_EPIT HELIAL_MESENCHYMAL_TRANSITION from Molecular Signatures Database v7.0 (MSigDB^3^) were selected as candidate EMT-related genes (Subramanian et al., 2005; Liberzon et al., 2015). The R package “sva” was applied to eliminate the batch effect among the datasets (Leek et al., 2019). DEEGs were identified in training cohort TCGA-STAD by using an R package “limma” (Ritchie et al., 2015). A false discovery rate (FDR) adjusted *p*-value < 0.05 and an absolute value of log_2_ (fold change) > 0.5 were considered as the criteria for DEEGs identification. Heatmaps were conducted by using R package “pheatmap” (Kolde, 2019).

### Construction of EMT-Related Gene Signature

Firstly, the prognostic DEEGs were screened out by using univariate Cox regression analyses for OS. Then LASSO regression was applied to construct a multi-gene signature with the prognostic DEEGs based on lambda.min. The optimal value of lambda was identified through tenfold cross-validations. Univariate Cox regression and LASSO regression were performed in R with “survival” and “glmnet” package (Therneau and Grambsch, 2000; Friedman et al., 2010; Therneau, 2020). Risk score of each patients was calculated based on the signature, using the formula as follows:

$$\mathrm{Risk}\,\mathrm{score}=\sum_{i=1}^{n}\beta \,i\times E\,x\,p\,i$$

in which the *Exp* represents the expression of gene and the β is the LASSO coefficient of gene. All samples were separated to high- and low-risk groups based on the optimal cut-off value determined by the “surv_cutpoint” function of the R package “survminer” (Kassambara et al., 2019), which uses the maxstat (maximally selected rank statistics) statistic to determine the optimal cutpoint for continuous variables.

### Assessment and Validation of EMT-Related Gene Signature

Receiver operating characteristic curve was performed to qualify the discrimination of the signature by measuring the area under the curve (AUC). ROC curve was plotted with R package “survivalROC” (Heagerty et al., 2000). Kaplan-Meier curve combined with a log-rank test for OS was performed to evaluate the predictive value of the signature by using the R package “survival” (Therneau and Grambsch, 2000; Therneau, 2020). Univariate and multivariate Cox regression were performed to identify whether risk score was an independent prognostic factor for OS. To validate the signature, the same methods were performed in the external validation cohort GSE62254.

### Correlation Between the Signature and Clinical Characteristics

To investigate the predictive ability of the prognostic signature in different clinical characteristics, all the patients were divided into subgroups according to age, gender, TNM stage, T stage, N stage, M stage and LNR. Survival analysis and investigation of risk score were performed in each subgroups. The relationship between risk levels and clinical characteristics was measured using chi-square test.

### Public Database Mining of Genes in EMT-Related Gene Signature

Oncomine^4^ was used to investigate the expression profile of the EMT-related gene in GC (Rhodes et al., 2007). The genomic alterations and co-expressed genes of the EMT-related gene were explored by using cBioportal^5^ (Gao et al., 2013). We used The Human Protein Atlas^6^ to study the expression profile of the EMT-related gene at a translational level (Uhlen et al., 2005, 2017). The networks between EMT-related genes and transcription factors (TFs) or miRNAs were predicted by NetworkAnalyst^7^ and drawn with Cytoscape 3.7.0 (Shannon et al., 2003; Lachmann et al., 2010; Hsu et al., 2014; Zhou et al., 2019). The prediction of TFs was based on ChEA database while the data of miRNAs prediction was collected from miRTarBase via NetworkAnalyst platform. Co-expressed genes with a Spearman correlation ≥ 0.4 or < −0.4 were submitted to Kyoto Encyclopedia of Genes and Genomes (KEGG) enrichment analysis by using R package “clusterProfiler” with a *p*-value < 0.05 and a *q*-value < 0.05 (Kanehisa and Goto, 2000; Yu et al., 2012). The enrichment analysis was visualized by R package “enrichplot” (Yu, 2019).

### Cell Culture

The human GC cell lines AGS and NCI-N87 and the human normal gastric mucosal cell line GES-1 were obtained from the Cell Bank of the Chinese Academy of Sciences (Shanghai, China). The human GC cell lines Hs-746T and SNU-719 were purchased from Procell (Wuhan, China). The human GC cell line SNU-5 was obtained from ATCC. Cells were cultured in RPMI-1640 medium (Gibco, Grand Island, NY, United States) supplemented with 10% fetal bovine serum (FBS) (NBCS) (PAA Laboratories, Inc., Pasching, Austria) at 37°C in an atmosphere of 5% CO2.

### RNA Isolation and Quantitative Real-Time PCR (qRT-PCR)

Total RNAs were extracted from cells by using Trizol reagent (Invitrogen, Carlsbad, CA, United States), and qRT-PCR was performed by using the PrimerScript RT Master Mix (Takara Bio, Inc., Shiga, Japan) and TB Green Premix Ex Taq (Takara Bio, Inc., Shiga, Japan) according to the manufacturer’s instructions. GAPDH was used as gene internal control and the final data were analyzed with the 2^–ΔΔCt^ method. The specific sense primers for ITGAV, DAB2, SERPINE1, MATN3, PLOD2 and GAPDH are listed in Supplementary Table 1.

### Establishment and Assessment of Signature-Based Nomogram

A nomogram for OS prediction was formulated based on the result of multivariate Cox regression by using the R package “rms” (Harrell, 2019). The C-index, ROC curve, calibration curve and DCA were used to assess the nomogram. The C-index and AUC of ROC curve were calculated to evaluate the discriminatory of the nomogram. The calibration curve was performed to compare the predicted survival outcome with the actual outcome by a bootstrap method with 1000 resamples. DCA was preformed to assess clinical utility of the nomogram by comparing the net benefit of the nomogram with all or none strategies (Vickers and Elkin, 2006). Akaike information criterion (AIC) was used to test the goodness of fit for models. The same methods were used to validate the results in the external validation cohort GSE62254.

### Gene Set Enrichment Analysis

Gene Set Enrichment Analysis (GSEA) was performed to study the different KEGG pathways between high- and low-risk groups in TCGA-STAD by using the GSEA software (v4.0.3^8^) (Subramanian et al., 2005). The reference sets for calculating Enrichment Score (ES) were c2.cp.kegg.v7.0.symbols.gmt. Gene sets were considered to be significantly enriched when *p*-value < 0.05 and FDR < 0.25 after performing 1000 permutations. The GSEA figures were plotted with the R package “ggplot2” (Wickham, 2016).

### Survival Analysis

The optimal cut-off value for high and low expression of gene or LNR was determined by the “surv_cutpoint” function of the R package “survminer” (Kassambara et al., 2019). Survival analysis was used to identify the difference of OS between the high and low expression groups of the EMT-related gene or the high- and low-risk groups of the signature. All the survival analyses mentioned above were performed by Kaplan-Meier curve with a two-side log-rank test.

### Statistical Analyses

All the analyses were performed in R v3.6.0 (The R Foundation for Statistical Computing, Vienna, Austria) and GraphPad Prism v7.00 (GraphPad Software Inc., United States). Boxplot was analyzed using Mann-Whitney U test. The R package “corrplot” was applied to draw the correlation plot of prognostic EMT-related genes and the Spearman test was used to analyze the correlation (Wei and Simko, 2017). Comparisons between qRT-PCR results from different cell lines were performed using one-way ANOVA and Dunnett’s T3 multiple comparison test and the results were presented as mean ± SD. *p* < 0.05 was considered statistically significant.

## Results

### Construction of the EMT-Related Gene Signature in GC

**FIGURE 1:**
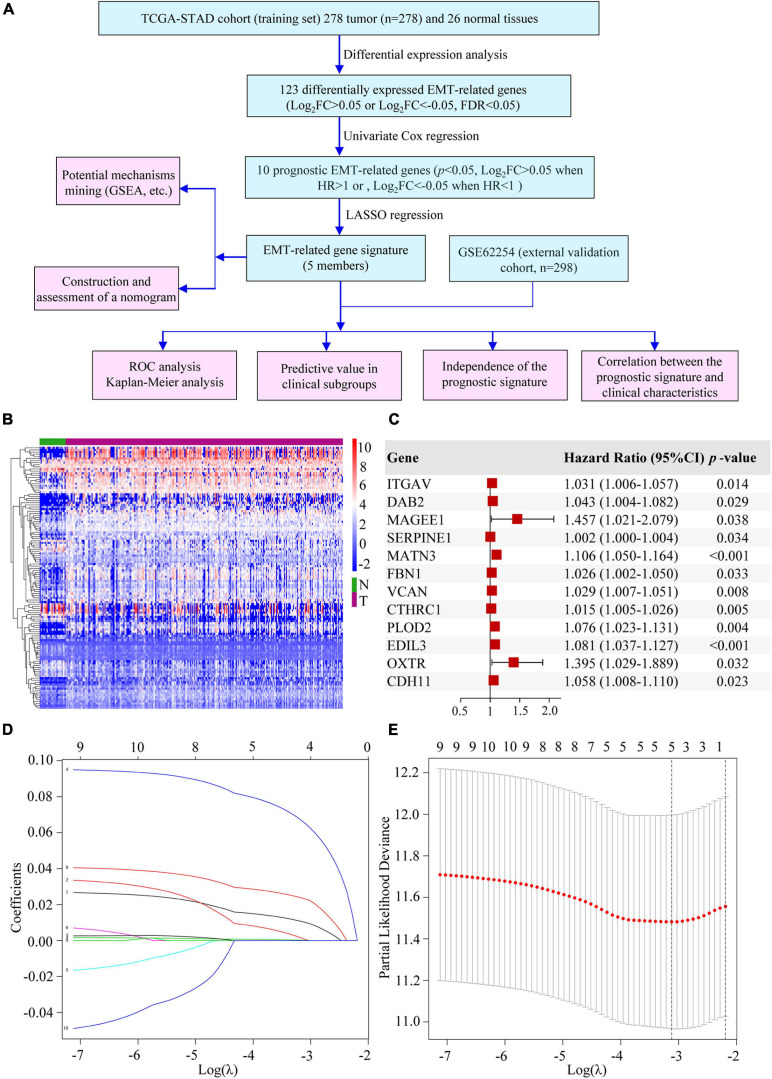
Establishment of the EMT-related gene signature. **(A)** The flow chart of our study. **(B)** Heatmap of DEEGs. **(C)** Univariate Cox regression analysis of DEEGs associated with OS in GC. **(D)** LASSO coefficient profiles of 10 prognostic genes of GC. **(E)** LASSO regression with ten-fold cross-validation obtained 5 prognostic genes by using the minimum λ.

We conducted our study as illustrated in Figure 1A. A total of 123 significantly differentially expressed EMT-related genes were identified from TCGA-STAD cohort, of which 89 were upregulated and 34 were downregulated in GC (Figure 1B and Supplementary Table 2). After an initial screening of EMT-related genes associated with OS by using univariate Cox regression analysis, 12 prognostic genes were found (Figure 1C). MAGEE1 and EDIL3 were excluded from our analysis because they were downregulated genes with HR > 1. Considering that there were correlations among 10 prognostic genes (Supplementary Figure 1), all of them were selected to the LASSO modeling to reduce multicollinearity (Figures 1D,E). The prognostic risk score of the signature was identified: risk score = 0.010325 × (expression level of ITGAV) + 0.000891 × (expression level of DAB2) + 0.000183 × (expression level of SERPINE1) + 0.065772 × (expression level of MATN3) + 0.023410 × (expression level of PLOD2). It was indicated that they were all risk factors for OS. The absolute values of coefficients indicated that MATN3 had the most influence on OS prediction, yet SERPINE1 had the least.

### Estimation and Validation of EMT-Related Gene Signature

**FIGURE 2:**
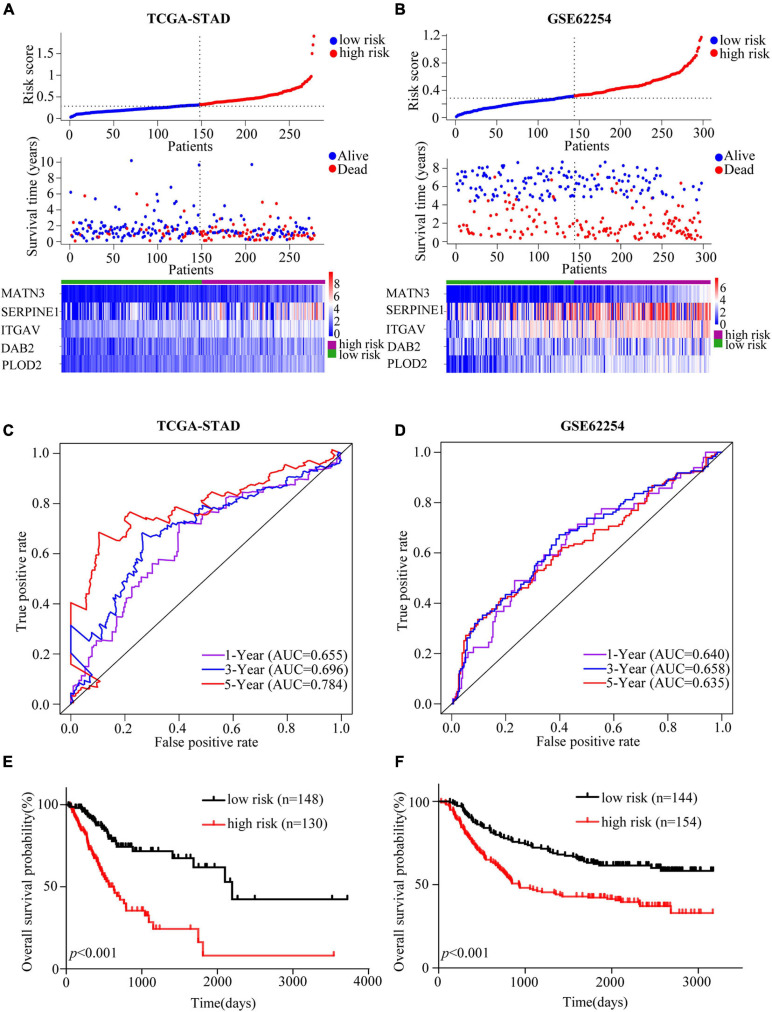
Assessment and validation of the EMT-related gene signature in GC. **(A,B)** Risk score analyses including risk score distributions, survival statuses and heatmaps of the EMT-related genes expression in the TCGA-STAD **(A)** and GSE62254 **(B)** cohorts. **(C,D)** Time-dependent ROC curves of the EMT-related gene signature in the TCGA-STAD **(C)** and GSE62254 **(D)** cohorts. **(E,F)** Kaplan-Meier estimates of OS based on the EMT-related gene signature in the TCGA-STAD **(E)** and GSE62254 **(F)** cohorts. The TCGA-STAD cohort was used as the training set while the cohort GSE62254 was used for external validation.

Based on the “surv_cutpoint” function of the R package “survminer,” we calculated that the optimal cut-off value was 0.318659. Patients in the TCGA cohort were divided into high- and low-risk groups according to the optimal cut-off. The patients’ risk score distribution, survival status and gene expression levels of EMT-related gene signature were presented in Figure 2A. To validate the predictive value of the EMT-related gene signature, risk scores for patients in GSE62254 were calculated with the same formula. And patients were separated into high- and low-risk groups according to the same cut-off. Risk score distribution, survival status, and gene expression levels of the signature were also shown (Figure 2B). The AUCs for 1-, 3-, and 5-year OS were 0.655, 0.696, and 0.784 in the TCGA cohort (Figure 2C). And in the validation cohort, the AUCs for 1-, 3- and 5-year OS were 0.640, 0.658, and 0.635, respectively, showing the good prognostic discrimination of the EMT-related gene signature (Figure 2D). The survival analysis showed that the OS of low risk group was better than that of high risk group (TCGA-STAD, *p* < 0.001; GSE62254, *p* < 0.001) (Figures 2E,F). Together, by modeling with training cohort and external validation, our results indicated that the EMT-related gene signature performed well for OS prediction.

### EMT-Related Gene Signature in Different Clinical Subgroups

**FIGURE 3:**
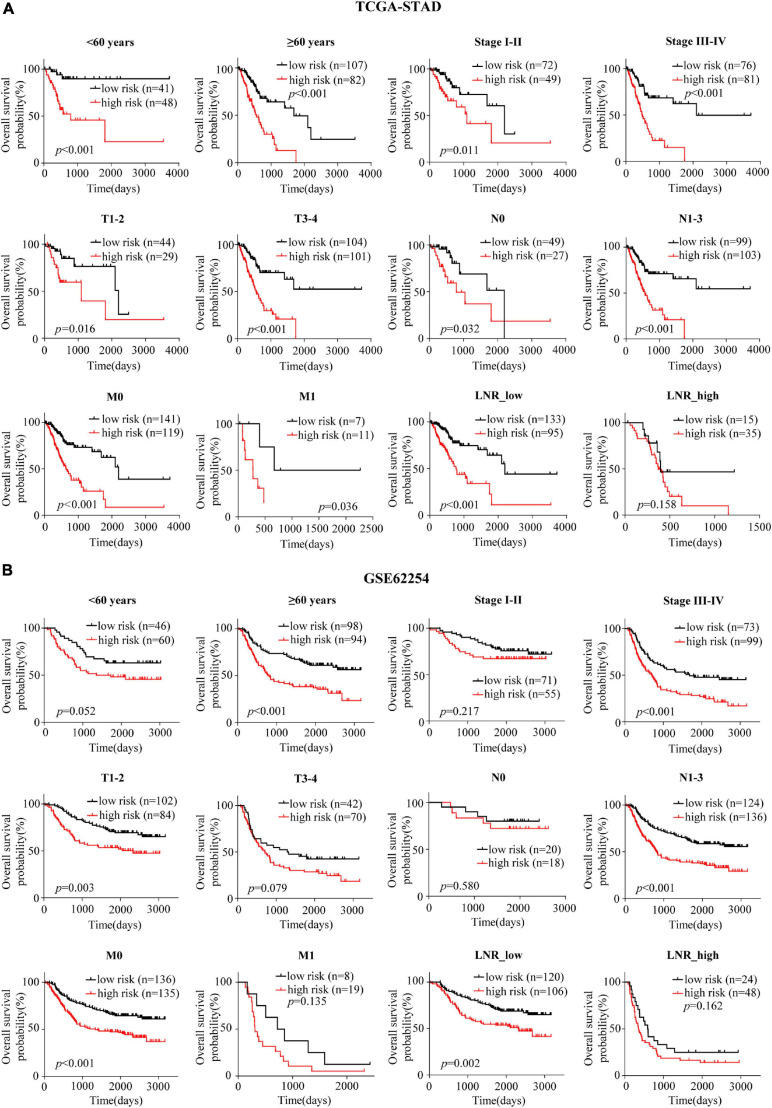
Confirmation of the signature for OS prediction in different clinical subgroups. **(A,B)** Kaplan-Meier estimates of OS based on the EMT-related gene signature in subgroups stratified according to age, stage, T, N, M, and LNR in the TCGA-STAD **(A)** and the GSE62254 **(B)** cohorts.

Patients were divided into different subgroups according to age, gender, TNM stage, T stage, N stage, M stage and LNR. Kaplan-Meier analyses of the EMT-related gene signature in subgroups showed that in the TCGA cohort, patients with high-risk had worse OS than patients with low-risk in < 60 years (*p* < 0.001), ≥ 60 years (*p* < 0.001), female (*p* < 0.001), male (*p* < 0.001), stage I-II (*p* = 0.011), stage III-IV (*p* < 0.001), T1-2 (*p* = 0.016), T3-4 (*p* < 0.001), N0 (*p* = 0.032), N1-3 (*p* < 0.001), M0 (*p* < 0.001), M1 (*p* = 0.036) and LNR_low (*p* < 0.001) subgroups (Figure 3A and Supplementary Figure 2A). Similar results could be obtained in subgroups such as ≥ 60 years (*p* < 0.001), female (*p* < 0.001), male (*p* = 0.005), stage III-IV (*p* < 0.001), T1-2 (*p* = 0.003), N1-3 (*p* < 0.001), M0 (*p* < 0.001), and LNR_low (*p* = 0.002) for the external validation cohort (Figure 3B and Supplementary Figure 2B). We performed univariate Cox regression based on the factors including age, stage, T, N, M, number of lymph nodes examined, number of positive nodes, LNR and risk score. As factors such as T, N and M are not independent of stage by definition and number of lymph nodes examined and number of positive nodes are components of LNR, they were omitted from multivariate Cox regression. So multivariate Cox regression was performed based on the factors including age, gender, stage, LNR and risk score (Supplementary Table 3). But, gender was finally excluded from our multivariate Cox regression model (shown in Tables 1, 2) as it was not a significant prognostic factor according to the multivariate Cox regression analysis in Supplementary Table 3. Univariate and multivariate Cox regression suggested that the five-gene prognostic signature was an independent OS predictor for GC in the TCGA (Table 1) cohort and the GEO cohort (Table 2).

**TABLE 1:** Univariate and multivariate Cox regression analyses of clinical characteristics and risk score in TCGA-STAD.

		**Univariate Cox**		**Multivariate Cox**	
		**regression**		**regression**	
**Characteristics**	**Number**	**Hazard Ratio (95%CI)**	***p*-value**	**Hazard Ratio (95%CI)**	***p*-value**
Age	278	1.016 (0.996–1.036)	0.121	1.029 (1.008–1.052)	0.008
Gender					
Male/Female	175/103	1.549 (0.988–2.429)	0.056		
Tumor stage					
II/I	82/39	1.349 (0.615–2.962)	0.455	1.182 (0.532–2.626)	0.681
III/I	127/39	1.953 (0.954–3.999)	0.067	1.258 (0.552–2.867)	0.585
IV/I	30/39	4.363 (1.984–9.595)	<0.001	3.050 (1.235–7.533)	0.016
T					
T3-4/T1-2	205/73	1.548 (0.951–2.521)	0.079		
N					
N1-3/N0	202/76	1.568 (0.958–2.566)	0.073		
M					
M1/M0	18/260	2.547 (1.317–4.925)	0.005		
Number of lymph nodes examined	278	0.994 (0.981–1.006)	0.323		
number of positive lymph nodes	278	1.053 (1.034–1.072)	<0.001		
LNR	278	4.548 (2.514–8.226)	<0.001	2.987 (1.412–6.322)	0.004
Risk score	278	4.447 (2.400–8.242)	<0.001	4.185 (2.039–8.588)	<0.001

**TABLE 2:** Univariate and multivariate Cox regression analyses of clinical characteristics and risk score in GSE62254.

		**Univariate Cox**		**Multivariate Cox**	
		**regression**		**regression**	
**Characteristics**	**Number**	**Hazard Ratio (95%CI)**	***p*-value**	**Hazard Ratio (95%CI)**	***p*-value**
Age	298	1.011 (0.996–1.026)	0.157	1.028 (1.012–1.044)	0.001
Gender					
Male/Female	197/101	0.917 (0.656–1.282)	0.611		
Tumor stage					
II/I	96/30	1.955 (0.759–5.034)	0.165	1.678 (0.649–4.338)	0.285
III/I	95/30	4.064 (1.623–10.176)	0.003	2.669 (1.030–6.920)	0.043
IV/I	77/30	9.737 (3.907–24.265)	<0.001	4.837 (1.755–13.328)	0.002
T					
T3-4/T1-2	112/186	2.365 (1.719–3.255)	<0.001		
N					
N1-3/N0	260/38	2.847 (1.450–5.589)	0.002		
M					
M1/M0	27/271	3.806 (2.460–5.888)	<0.001		
Number of lymph nodes examined	298	0.987 (0.976–0.998)	0.023		
number of positive lymph nodes	298	1.072 (1.056–1.087)	<0.001		
LNR	298	25.430 (13.312–48.578)	<0.001	7.030 (2.803–17.631)	<0.001
Risk score	298	5.205 (2.750–9.853)	<0.001	4.059 (2.097–7.855)	<0.001

### Correlation Between the EMT-Related Gene Signature and Clinical Characteristics

**FIGURE 4:**
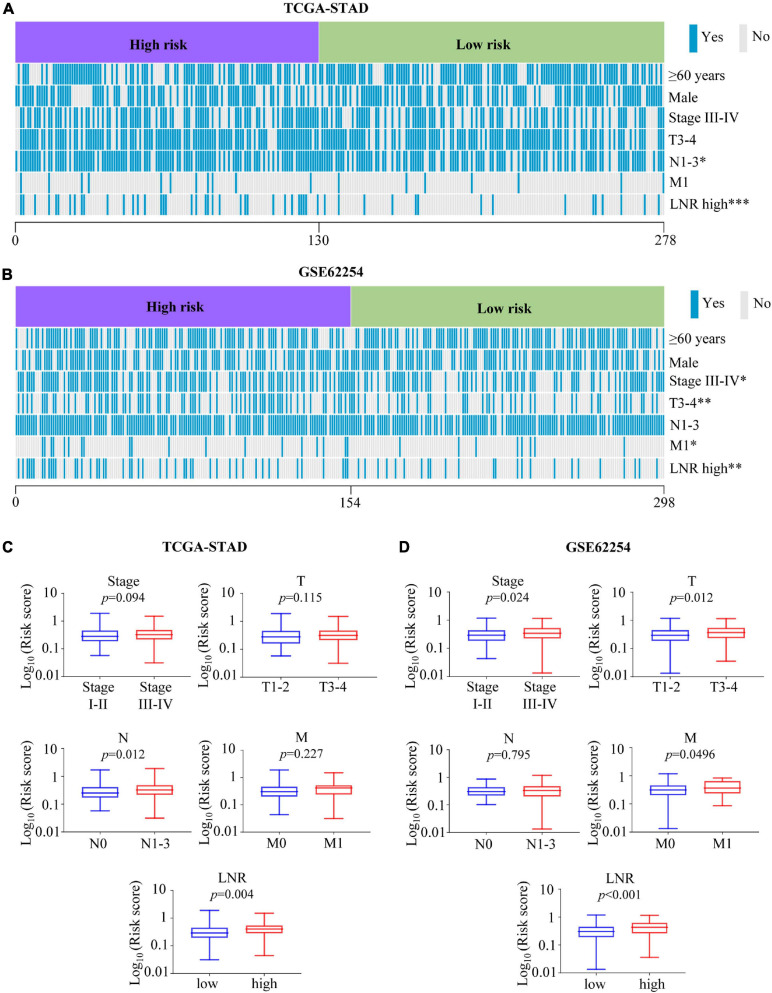
Association between the risk score with clinical characteristics. **(A,B)** Heatmaps of association between the risk score with clinical characteristics in the TCGA-STAD **(A)** and GSE62254 **(B)** cohorts. **(C,D)** Comparisons of risk score among different clinical subgroups stratified based on stage, T, N, M, and LNR in the TCGA-STAD **(C)** cohort and GSE62254 **(D)** cohort. ^∗^*p* < 0.05, ^∗∗^*p* < 0.01, ^∗∗∗^*p* < 0.001.

Distribution of several clinical parameters varied between high- and low-risk groups. It was illustrated that there were more N1-3 or LNR_high cases in the high-risk group than in the low-risk group of the TCGA cohort (N, *p* = 0.021; LNR, *p* < 0.001) (Figure 4A and Supplementary Table 4). In the validation cohort, more stage III-IV, T3-4, M1 or LNR_high cases could be found in the high-risk group (stage, *p* = 0.018; T, *p* = 0.004; M, *p* = 0.042; LNR, *p* = 0.003) (Figure 4B and Supplementary Table 5). What’s more, in the TCGA cohort, patients with N1-3 (*p* = 0.012), LNR_high (*p* = 0.004) tended to have higher risk scores (Figure 4C). And patients with stage III-IV (*p* = 0.024), T3-4 (*p* = 0.012), M1 (*p* = 0.0496), LNR_high (*p* < 0.001) yielded higher risk scores in the GEO cohort (Figure 4D).

### Expression Profiles and Survival Analyses of the Five Members

**FIGURE 5:**
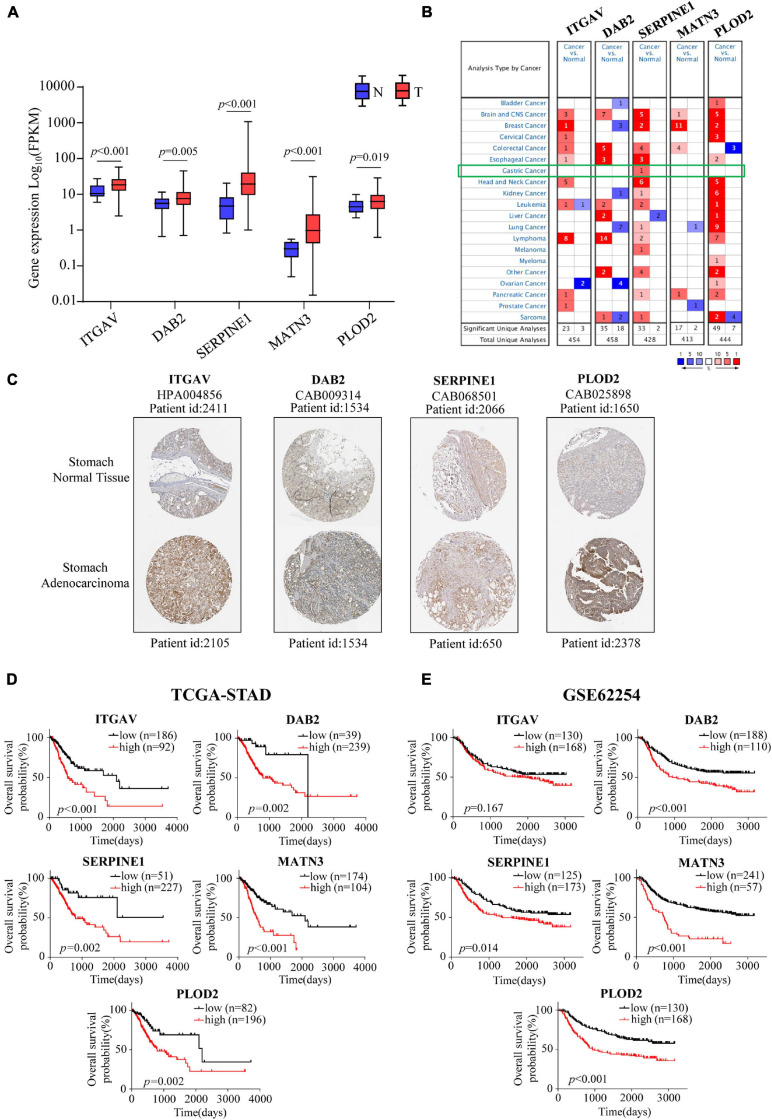
Expression profiles, Kaplan-Meier analyses of EMT-related signature members. **(A)** The mRNA expression profiles of members in EMT-related signature in the TCGA-STAD cohort. **(B)** Expression profiles of EMT-related signature members in Oncomine database. **(C)** The protein expression profiles of EMT-related signature members in the Human Protein Atlas database. **(D,E)** Kaplan-Meier estimates of OS based on EMT-related signature members in the TCGA-STAD cohort **(D)** and the GSE62254 cohort **(E)**.

**FIGURE 6:**
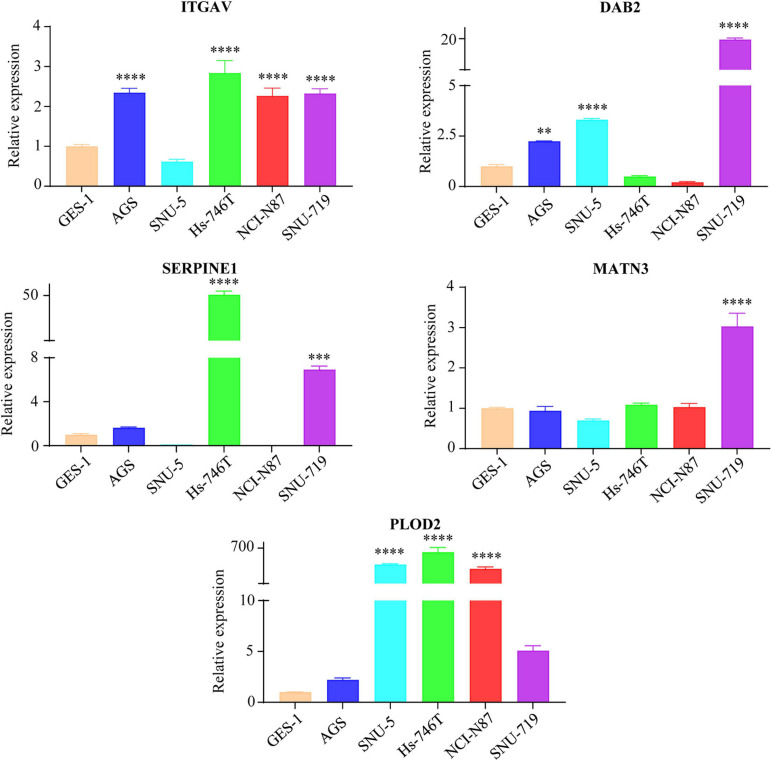
The mRNA expression levels of EMT-related genes (ITGAV, DAB2, SERPINE1, MATN3, and PLOD2) in GC cell lines and normal gastric mucosal cell line GES-1 detected by qRT-PCR. The gastric mucosa epithelial cell line GES-1 was used as control. ^∗∗^*p* < 0.01, ^∗∗∗^*p* < 0.001, and ^****^p < 0.0001.

To make a complete analysis of the contributions of EMT-related gene signature members in GC, the expression profiles and OS predictive values of ITGAV, DAB2, SERPINE1, MATN3 and PLOD2 were investigated. As shown in Figure 5A, all of them were significantly upregulated in GC samples in the TCGA cohort. The EMT-related gene signature members were retrieved using the Oncomine database. It was indicated that EMT-related gene signature members acted as oncogenes in most types of cancer (Figure 5B). There was 1 dataset for SERPINE1 suggesting its upregulation in GC. The images of immunohistochemistry (IHC) staining showed the protein expression of IAGAV, DAB2, SERPINE1 and PLOD2 in GC (Figure 5C). However, we did not find the protein expression images of MATN3 in the database. In the TCGA cohort, all of the EMT-related gene signature members were significantly associated with unfavorable OS outcome (Figure 5D) (ITGAV, *p* < 0.001; DAB2, *p* = 0.002; SERPINE1, *p* = 0.002; MATN3, *p* < 0.001; PLOD2, *p* = 0.002). Similarly, all the genes except ITGAV (*p* = 0.167) were observed as significantly unfavorable prognostic genes in GSE62254 (Figure 5E) (DAB2, *p* < 0.001; SERPINE1, *p* = 0.014; MATN3, *p* < 0.001; PLOD2, *p* < 0.001). Furthermore, we detected the mRNA levels of the EMT-related genes in human GC cell lines (AGS, SNU-5, Hs-746T, NCI-N87, SNU-719) and normal gastric mucosal cell line GES-1. Elevated expression of ITGAV was found in AGS, Hs-746T, NCI-N87 and SNU-719 compared with GES-1. The expression of DAB2 in AGS, SNU-5 and SNU-719 was higher than that in GES-1. The expression level of SERPINE1 in Hs-746T and SNU-719 and the expression of MATN3 in SNU-719 were 2-fold higher than those in GES-1. Expression of PLOD2 was significantly higher in SNU-5, Hs-746T and NCI-N87 compared with GES-1 (Figure 6).

### Potential Mechanisms Mining of the Five Members

**FIGURE 7:**
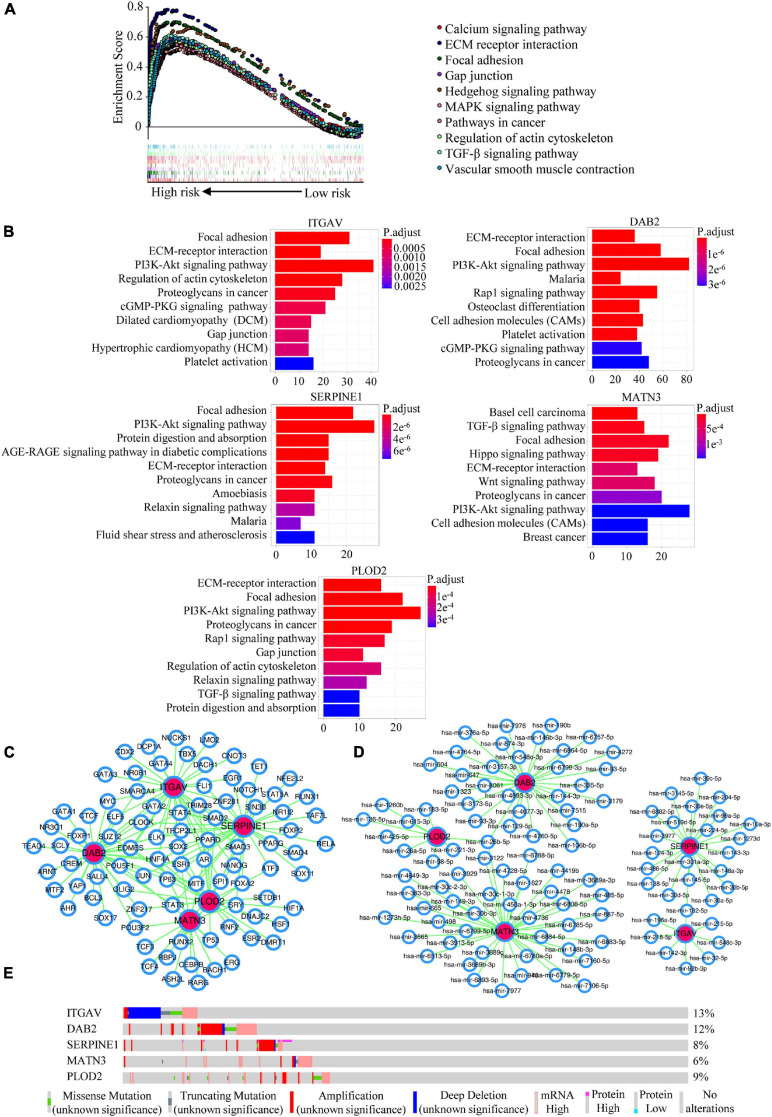
Potential mechanisms mining of the EMT-related signature. **(A)** GSEA analysis for the EMT-related signature. **(B)** KEGG enrichment analyses for co-expressed genes with EMT-related signature members. **(C)** The predicted network of TFs and EMT-related signature members in the NetworkAnalyst database. **(D)** The predicted networks of miRNAs and EMT-related signature members in the NetworkAnalyst database. **(E)** The genomic alterations of EMT-related signature members in the cBioportal database.

In order to explore the potential mechanisms of the five members in GC, we conducted the GSEA analysis for the prognostic signature, and investigated the enriched KEGG pathways, TFs/miRNA predictions, genomic alterations of the EMT-related signature members. GESA analysis revealed that the high-risk group might be involved in KEGG pathways such as calcium signaling pathway, ECM receptor interaction, focal adhesion, gap junction and other pathways (Figure 7A). What’s more, the KEGG enrichment analyses for co-expressed genes associated with EMT-related signature members were presented in Figure 7B. The KEGG pathways such as focal adhesion, ECM-receptor interaction, PI3K-Akt signaling pathway and proteoglycans in cancer were the top significant pathways related to all the members. It was interesting that focal adhesion and ECM-receptor interaction were the two pathways that both appeared in the results of GSEA and pathway enrichment analysis, which we thought were important to our signature. The TFs and miRNAs connected with EMT-related gene signature members were investigated by NetworkAnalyst (Figures 7C,D). Explored by using the cBioportal database, the genomic alterations of EMT-related gene signature members in GC varied from 6 to 13% (Figure 7E) (ITGAV, 13%; DAB2, 12%; SERPINE1, 8%; MATN3, 6%; PLOD2, 9%).

### Construction and Validation of the Signature-Based Nomogram

**FIGURE 8:**
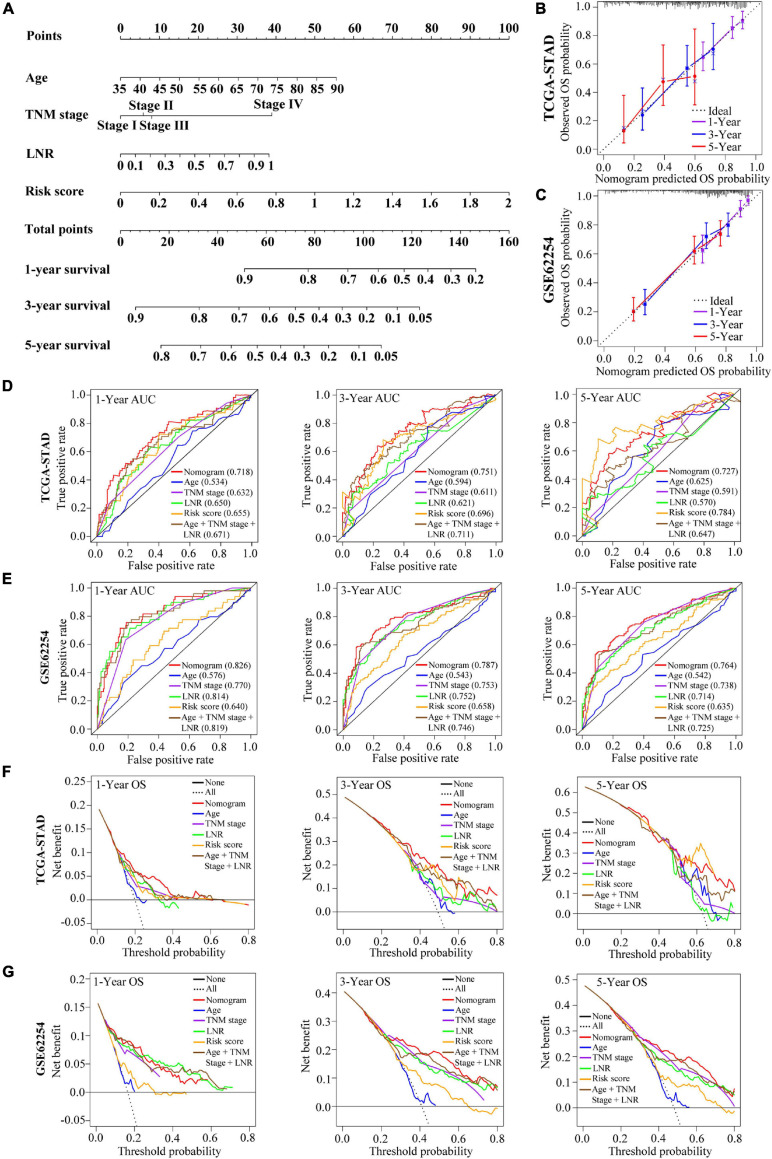
Construction and validation of a nomogram for OS prediction in GC. **(A)** The nomogram consisting of age, TNM stage, LNR and the risk score. **(B,C)** Calibration curves of the nomogram for 1-, 3-, and 5-year OS prediction in the TCGA cohort **(B)** and the GEO cohort **(C)**. The X-axis represents the nomogram-predicted outcome while Y-axis represents the observed outcome. The 45° line represents the best prediction. **(D,E)** Time-dependent ROC curves of the nomogram, age, TNM stage, LNR, risk score and age + TNM stage + LNR model for 1-, 3-, and 5-year OS prediction in the TCGA cohort **(D)** and the GEO cohort **(E)**. **(F,G)** DCA curves of the nomogram, age, TNM stage, LNR, risk score and age + TNM stage + LNR model for 1-, 3-, and 5-year OS prediction in the TCGA cohort **(F)** and the GEO cohort **(G)**. The plots show the expected net benefits at different threshold probability. The black line “None” represents the assumption that event will happen in no patients while the dash line “All” represents the assumption that event will happen in all patients.

A nomogram integrating the risk score, age, TNM stage and LNR for OS prediction of the patients with GC was shown in Figure 8A. The nomogram was built based on the variables applied to the final multivariable Cox regression of the training cohort above (Table 1). The C-index, ROC curve, calibration curve and DCA were used to quantify the model’s discrimination, calibration and clinical usefulness.

In the training cohort, the C-index of the nomogram for OS prediction was 0.702 and the AUCs of the ROC for 1-, 3- or 5-year OS of the nomogram were 0.718, 0.751, and 0.727, respectively (Figure 8D). Compared with age, TNM stage, LNR and the age + TNM stage + LNR model, the combined nomogram yielded largest AUC for 1-, 3-year OS but not for 5-year OS. The validation cohort GSE62254 was used to test the predictive accuracy of the nomogram. The C-index in validation cohort was 0.730 and the AUC values of ROC were 0.826 at 1 year, 0.787 at 3 years and 0.764 at 5 years (Figure 8E). We could find that in the validation cohort, the AUCs of the nomogram (age + TNM + LNR + risk score model) were better than age + TNM + LNR model, or age, TNM stage, LNR alone for 1-, 3- and 5-year OS, suggesting that the nomogram presented better discrimination when including risk score into model for prognostication in GC to a certain extent, and we could explain the modest improvement by the EMT risk score. The calibration curve and DCA curves of the training cohort TCGA-STAD were presented in Figures 8B,F. In the validation cohort GSE62254, the calibration curves for the probabilities of 1-, 3- or 5-year OS demonstrated good agreement between prediction by nomogram and actual observation (Figure 8C). Shown by the DCA curves of validation set, the combined nomogram yielded modest additional net benefit for 3- or 5-year OS probability from using the nomogram instead of clinical model without EMT risk score, illustrating that the combined nomogram had potential for clinical utility and the modest additional net benefit for 3- or 5-year OS probability might be explained by the EMT risk score (Figure 8G). We used AIC to test the goodness of fit for models including our nomogram (using age, TNM stage and LNR as well as EMT risk score) and the models combining two or three of the factors including age, TNM stage, LNR and risk score (Supplementary Table 6). We could find that the nomogram might be the optimal model based on the smallest value for the AIC statistic. Collectively, our combined nomogram performed well for OS prediction in GC.

## Discussion

Gastric cancer remains a great challenge for public health worldwide and its OS is still not satisfactory. More and more attention was paid to the role of EMT in OS prediction (Tan et al., 2014; Cao et al., 2020). It has been revealed that EMT could lead to drug resistance in breast cancer, lung cancer and GC, and metastasis in bladder cancer and GC, which may be the reasons why EMT could contribute to a worse OS (Huang J. et al., 2015; Tao et al., 2020; Tian et al., 2020; Wang et al., 2020; Zhang et al., 2020). Many single potential prognostic genes that associated with EMT in GC have been reported by researchers. But as we know, EMT is a complex process that is triggered by many genes. So integration of these genes tends to be significant for understanding the process of EMT. Thanks to the rapid improvements in sequencing techniques, mining the gene signatures from transcriptional profiles for individual risk stratification of patients with cancer has flourished. Combining the gene signature with clinical parameters has been highlighted when predicting the survival and considering individualized treatment for patients.

Similar to our study, Cao et al. have built an EMT-related gene signature that might facilitate risk stratification of patients and personalized treatment in bladder cancer (Cao et al., 2020). Besides, Tan et al. developed a generic EMT signature to estimate extent of EMT in several kinds of tumors, showing that EMT is linked to OS in ovarian cancer, glioblastoma and GC, but given that GC is a specific cancer different from others, a signature specific to GC may be a better choice for OS prediction (Tan et al., 2014). Zhu et al. constructed another prognostic and predictive classifier for GC, and intriguingly, the high-score group was related to EMT subtype, suggesting the importance of EMT in risk stratification (Zhu et al., 2018). Therefore, a comprehensive signature of EMT-related gene was necessary to be built for outcome prediction of GC patients.

In this study, we developed a novel five-gene signature related to EMT which included ITGAV, DAB2, SERPINE1, MATN3 and PLOD2. EMT is a complicated and sophisticated biological process involving many pathways. The GSEA and pathway enrichment analysis in our study revealed that focal adhesion and ECM-receptor interaction might be the two important significantly enriched EMT-related pathways to the signature. Focal adhesion signaling events play essential roles in reorganizing the actin cytoskeleton, changing cell shape and motility, and regulating cell proliferation, differentiation and survival (Petit and Thiery, 2000). Extracellular matrix (ECM), constituting the main part of the extracellular microenvironment, can directly interact with cells, regulating cell growth, migration, proliferation, differentiation, metabolism, and function by integrin or other cell surface receptors (Yang et al., 2020). The results of GSEA and pathway enrichment analysis suggested the potential mechanisms involved in our signature, providing us direction for further experiment research in the future. All of members in the signature were negative predictors of OS in our signature, and they all have been reported in cancers. ITGAV belongs to the integrin family of extracellular matrix receptors, functioning in cell surface adhesion and signaling. Suppression of ITGAV inhibited cell growth, invasion, and self-renewal of breast cancer by altering BCL2 and PXN levels (Cheuk et al., 2020). Evidences have been reported that it could promote growth, migration, and invasion of GC cells, and was positively associated with lymph node metastasis (Wang et al., 2019a). DAB2, initially known as DOC-2, was considered to be a tumor suppressor because of its absence in 85% of ovarian cancer (Fazili et al., 1999). However, Chao et al. suggested that upregulation of DAB2 could promote EMT by inhibiting E-cadherin while stimulating vimentin and phospho-FAK, indicating the significance of DAB2 in EMT (Chao et al., 2012). In human gastric carcinomas, DAB2^+^ tumor-associated macrophages correlated with a poor clinical outcome (Marigo et al., 2020). SERPINE1, an inhibitor of tissue plasminogen activator and urokinase, is a fibrinolytic inhibitor. It was validated that SERPINE1 could promote migration and invasion by regulating EMT in GC (Yang et al., 2019). What’s more, it was identified as prognostic biomarker for GC by bioinformatics, consistent with our study (Li et al., 2019; Xu et al., 2019). MATN3 encodes a protein which belongs to von Willebrand factor A domain containing protein family related to the formation of filamentous networks in the extracellular matrices of various tissues (Wagener et al., 1997). It was verified that MATN3 protein was upregulated in gastric adenocarcinoma, acting as a predictor of poor prognosis (Wu et al., 2018). MATN3 has been used for previous prognostic models to predict recurrence for GC patients, indicating the vital performance of MATN3 in GC (Lee et al., 2014; Zhou et al., 2018). But the mechanism of MATN3 in GC is not yet clear. PLOD2 is a kind of enzyme that catalyzes the hydroxylation of lysyl residues in collagen-like peptides (Qi and Xu, 2018). PLOD2 has been shown to promote metastasis in cancer such as breast cancer, biliary tract cancer and lung cancer (Du et al., 2017; He et al., 2018; Okumura et al., 2018), etc. Besides, PLOD2 was reported to play an important role in peritoneal dissemination of GC, and it was regulated by hypoxia-inducible factor-1 (HIF-1) and involved in extracellular matrix remodeling, alignment and mechanical properties (Kiyozumi et al., 2018). According to the investigations above, five genes in the EMT-related signature have an important impact on the carcinogenesis and tumor progression. In our study, by using qRT-PCR assays, we could find the high mRNA expression of the five EMT-related genes in the GC cell lines. Probably, if the mechanisms of five genes in GC process are explored deeply and widely, they can better serve as biomarkers for GC.

The EMT-related gene signature with five prognostic genes was constructed by applying univariate Cox regression and LASSO regression. LASSO regression is a method which can reduce the risk of overfitting in the model, and it was used to improve the predictor selection in our signature. Then, patients were divided into high- and low-risk groups based on the signature. With external validation, the ROC curve and survival analysis showed that the signature performed well and the high-risk patients had poorer OS. Univariate and multivariate Cox regression indicated that the signature could be an independent factor to predict OS. In order to improve the signature’s ability of OS prediction, we built a nomogram which combined the signature with clinical parameters according to the variables of multivariate Cox regression above. And it was assessed by C-index, ROC curve, calibration curve, and DCA. External validation was conducted to verify the prognostic value of the combined nomogram. In validation cohort, ROC curves showed that the nomogram (using age, TNM stage, and LNR as well as EMT risk score) had a better discrimination than age + TNM stage + LNR model without EMT risk score. Prefect agreement could be seen when comparing predictive survival outcome with the actual outcome in the calibration curve. DCA curves indicated that the nomogram might have good clinical usefulness for 3-, 5-year OS prediction and the modest additional net benefit for 3- or 5-year OS probability from using the nomogram instead of clinical model without EMT risk score might be explained by the EMT risk score. What’s more, based on the smallest value for the AIC statistic, the nomogram (combining age, TNM stage, LNR and EMT risk score) might be the optimal model. Thus, combing the prognostic signature and clinical characteristics may improve prognostication for GC to some extent, suggesting the prognostic signature’s and nomogram’s potential application values for individual risk stratification in clinic. What’s more, it provides a new perspective for covering the insufficiency of current staging system.

Several limitations should also be noticed in our study. Firstly, our study was a retrospective study based on two public datasets in which most patients are Asian and White, and because of geographically variation, extending our findings to more other ethnic cohorts is necessary. Secondly, though evidences were provided by our study that the five-gene signature was a significantly predictor for GC survival, underlying mechanisms between genes of the signature and GC are not clear enough. Further experiment researches of five-gene signature in lab are crucial before clinical use. Thirdly, more independent cohorts are needed to validate the prognostic signature and nomogram. Fourthly, TCGA-STAD dataset recorded cases’ original staging, which, over time, reflected AJCC different editions. Because of the incomplete detailed descriptions for staging, the standardization for TNM staging was difficult. We hope that this concern will be resolved in the future for more accurate modeling. Fifthly, resection quality at the time of surgery is an important prognostic factor in GC, but insufficient information on resection quality of cohorts in our study resulted in our omission with this consideration. Thus, further well-designed, prospective, international studies are necessary to verify our findings.

In summary, EMT is vital to malignant progression and associated with poor OS of patients with GC. Here, we identified an EMT-related gene signature and a combined nomogram to predict OS of GC, which can add clinical value to traditional staging system for predicting OS, and might facilitate individualized treatment and clinical decision-making for GC patients.

## Data Availability Statement

Publicly available datasets were analyzed in this study. This data can be found here: TCGA database: https://portal.gdc.cancer.gov/; GEO database: https://www.ncbi.nlm.nih.gov/geo/.

## Ethics Statement

Ethical review and approval was not required for the study on human participants in accordance with the local legislation and institutional requirements. Written informed consent for participation was not required for this study in accordance with the national legislation and the institutional requirements.

## Author Contributions

JW, LX, and XW designed and conceived this study. WD, YX, and JZ collected and analyzed the data. WT, JLin, LH, MP, JLi, and SL performed statistical analyses. WD and YX contributed to writing and revised the manuscript. All authors read and approved the final manuscript.

## Conflict of Interest

The authors declare that the research was conducted in the absence of any commercial or financial relationships that could be construed as a potential conflict of interest.

## Footnotes

1 https://portal.gdc.cancer.gov/

2 https://www.ncbi.nlm.nih.gov/geo/

3 https://www.gsea-msigdb.org/gsea/msigdb/index.jsp

4 https://www.oncomine.org/

5 https://www.cbioportal.org/

6 https://www.proteinatlas.org/

7 https://www.networkanalyst.ca/

8 https://www.gsea-msigdb.org/gsea/index.jsp

## Supplementary Material

Supplementary Figure 1 Correlation plot of 10 prognostic EMT-related genes in the TCGA cohort.

Supplementary Figure 2 Confirmation of the signature for OS prediction in clinical subgroups stratified according to gender. **(A,B)** Kaplan-Meier estimates of OS based on the EMT-related gene signature in subgroups classified by gender in the TCGA-STAD **(A)** and the GSE62254 **(B)** cohorts.

Supplementary Table 1 List of the primers used for qRT-PCR.

Supplementary Table 2 123 significantly differentially expressed EMT-related genes.

Supplementary Table 3 Multivariate Cox regression of TCGA-STAD and GSE62254 cohorts with variables including age, gender, stage, LNR and risk score.

Supplementary Table 4 Clinical characteristics of TCGA-STAD cohort patients in different risk groups.

Supplementary Table 5 Clinical characteristics of GSE62254 cohort patients in different risk groups.

Supplementary Table 6 The profiles of the EMT-related gene signature based nomogram and models with different factors’ combinations.
