# Supplementary figures and images for: Identification of an EMT-Related Gene Signature for Predicting Overall Survival in Gastric Cancer
Source: Front Genet. 2021 Jun 24;12:661306. doi: 10.3389/fgene.2021.661306 (PMC8264558; doi:10.3389/fgene.2021.661306)

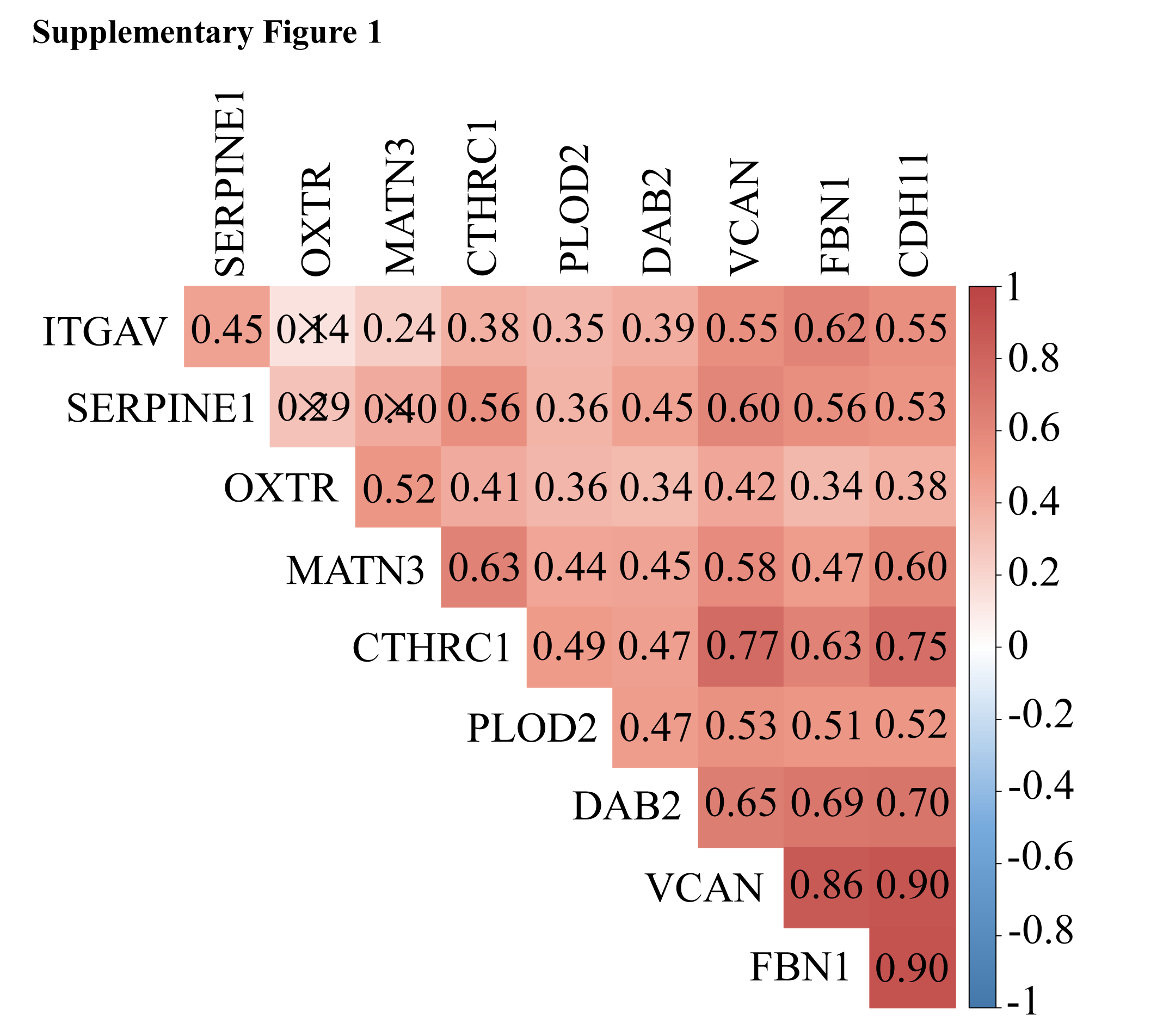

Supplement: Supplementary Figure 1 — Correlation plot of 10 prognostic EMT-related genes in the TCGA cohort. [file Image_1.TIF]

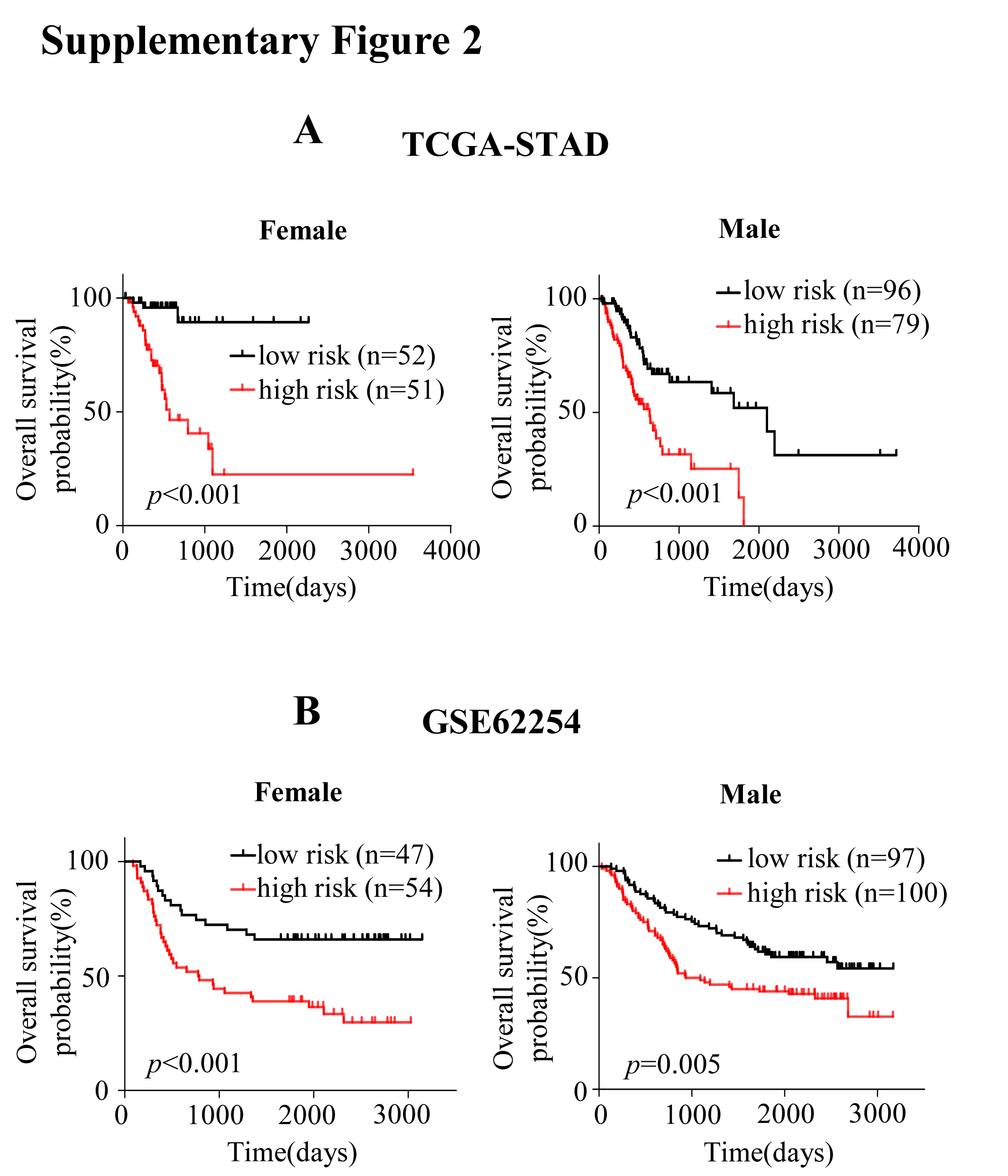

Supplement: Supplementary Figure 2 — Confirmation of the signature for OS prediction in clinical subgroups stratified according to gender. (A,B) Kaplan-Meier estimates of OS based on the EMT-related gene signature in subgroups classified by gender in the TCGA-STAD (A) and the GSE62254 (B) cohorts. [file Image_2.TIF]
