# Supplementary material for: Identification of an EMT-Related Gene Signature for Predicting Overall Survival in Gastric Cancer
Source: Front Genet. 2021 Jun 24;12:661306. doi: 10.3389/fgene.2021.661306 (PMC8264558; doi:10.3389/fgene.2021.661306)
Supplement: Supplementary Table 1 — List of the primers used for qRT-PCR. [file Table_1.DOCX]

**Supplementary Table 1.** List of the primers used for qRT-PCR.

| **Name** | **Position or orientation** | **Sequence (5’-3’)** |
| --- | --- | --- |
| ITGAV | F | ATCTGTGAGGTCGAAACAGGA |
|  | R | TGGAGCATACTCAACAGTCTTTG |
| DAB2 | F | GTAGAAACAAGTGCAACCAATGG |
|  | R | GCCTTTGAACCTTGCTAAGAGA |
| SERPINE1 | F | ACCGCAACGTGGTTTTCTCA |
|  | R | TTGAATCCCATAGCTGCTTGAAT |
| MATN3 | F | TCTCCCGGATAATCGACACTC |
|  | R | CAAGGGTGTGATTCGACCCA |
| PLOD2 | F | CATGGACACAGGATAATGGCTG |
|  | R | AGGGGTTGGTTGCTCAATAAAAA |
| GAPDH | F | AAATCCCATCACCATCTTCC |
|  | R | TCACACCCATGACGAACA |
