# Supplementary material for: Identification of an EMT-Related Gene Signature for Predicting Overall Survival in Gastric Cancer
Source: Front Genet. 2021 Jun 24;12:661306. doi: 10.3389/fgene.2021.661306 (PMC8264558; doi:10.3389/fgene.2021.661306)
Supplement: Supplementary Table 2 — 123 significantly differentially expressed EMT-related genes. [file Table_2.DOCX]

**Supplementary Table 2.** 123 significantly differentially expressed EMT-related genes

| **Gene** | **LogFC** | ***p*-value** | **FDR** |
| --- | --- | --- | --- |
| CTHRC1 | 13.723826 | 1.89E-13 | 4.91E-12 |
| COMP | 12.51072736 | 6.15E-06 | 1.75E-05 |
| MMP3 | 10.99512993 | 6.98E-06 | 1.95E-05 |
| SPP1 | 7.562781637 | 1.66E-07 | 6.18E-07 |
| CXCL8 | 6.75843851 | 1.43E-07 | 5.55E-07 |
| BGN | 6.24911827 | 5.87E-12 | 7.63E-11 |
| LAMC2 | 6.055589113 | 2.68E-12 | 4.07E-11 |
| SERPINE1 | 5.639119153 | 3.21E-09 | 1.95E-08 |
| PMEPA1 | 5.348123028 | 1.94E-11 | 1.96E-10 |
| SFRP4 | 5.174344258 | 2.71E-06 | 7.96E-06 |
| INHBA | 5.019866862 | 2.93E-14 | 1.78E-12 |
| COL3A1 | 4.901129475 | 8.00E-10 | 5.82E-09 |
| TNFRSF11B | 4.705675553 | 2.49E-08 | 1.08E-07 |
| THBS2 | 4.650740379 | 4.62E-08 | 1.91E-07 |
| COL11A1 | 4.614196713 | 4.62E-13 | 9.34E-12 |
| COL1A2 | 4.402962943 | 1.50E-09 | 9.74E-09 |
| CXCL1 | 4.373462792 | 3.83E-07 | 1.37E-06 |
| COL5A2 | 3.828367794 | 3.15E-12 | 4.41E-11 |
| MMP1 | 3.497061675 | 2.17E-05 | 5.73E-05 |
| MEST | 3.454154493 | 2.88E-13 | 6.56E-12 |
| TIMP1 | 3.365336712 | 1.54E-11 | 1.65E-10 |
| MXRA5 | 3.294952989 | 2.93E-09 | 1.84E-08 |
| SPARC | 3.173419536 | 6.28E-10 | 4.97E-09 |
| CXCL6 | 3.1451839 | 1.53E-07 | 5.78E-07 |
| SERPINH1 | 2.846138093 | 6.67E-14 | 3.04E-12 |
| VCAN | 2.760731196 | 6.28E-09 | 3.57E-08 |
| MATN3 | 2.713047453 | 9.57E-09 | 4.90E-08 |
| THY1 | 2.702512539 | 1.45E-09 | 9.74E-09 |
| PLOD3 | 2.661291009 | 1.16E-13 | 3.61E-12 |
| COL5A1 | 2.593616352 | 4.96E-07 | 1.74E-06 |
| COL12A1 | 2.562704464 | 2.63E-08 | 1.12E-07 |
| PDGFRB | 2.499048222 | 8.85E-09 | 4.73E-08 |
| MCM7 | 2.444523477 | 9.35E-12 | 1.10E-10 |
| MMP14 | 2.436608661 | 1.94E-10 | 1.68E-09 |
| ADAM12 | 2.349468894 | 8.42E-15 | 7.67E-13 |
| LOX | 2.348369831 | 9.69E-09 | 4.90E-08 |
| COL4A1 | 2.271572326 | 1.20E-09 | 8.41E-09 |
| IGFBP3 | 2.181020987 | 1.90E-06 | 6.07E-06 |
| LOXL2 | 2.150164268 | 2.47E-10 | 2.05E-09 |
| PVR | 2.102082754 | 9.66E-12 | 1.10E-10 |
| BMP1 | 2.014081426 | 1.19E-13 | 3.61E-12 |
| ITGA2 | 1.956316385 | 1.66E-08 | 7.56E-08 |
| COLGALT1 | 1.932650427 | 5.02E-15 | 7.67E-13 |
| LRRC15 | 1.88870029 | 0.000331364 | 0.00068532 |
| VEGFA | 1.871288555 | 6.77E-09 | 3.73E-08 |
| TNFRSF12A | 1.866823208 | 1.44E-08 | 6.73E-08 |
| COL6A3 | 1.853766824 | 2.43E-06 | 7.36E-06 |
| LAMA3 | 1.836372066 | 0.002150565 | 0.003727647 |
| FSTL3 | 1.769324847 | 0.000173068 | 0.00039373 |
| CDH11 | 1.690228534 | 1.42E-06 | 4.78E-06 |
| COL7A1 | 1.645534302 | 5.12E-09 | 3.01E-08 |
| COL5A3 | 1.531877295 | 1.37E-07 | 5.42E-07 |
| NNMT | 1.500834859 | 0.002070679 | 0.003623688 |
| PLAUR | 1.463748165 | 3.71E-06 | 1.07E-05 |
| FAP | 1.458131485 | 2.31E-12 | 3.82E-11 |
| MSX1 | 1.436142256 | 1.57E-06 | 5.18E-06 |
| TGFBI | 1.407855848 | 0.002765994 | 0.004661212 |
| PRRX1 | 1.367001619 | 0.000153437 | 0.00035802 |
| LUM | 1.366518075 | 0.003484818 | 0.00576579 |
| IL32 | 1.323880148 | 0.000218463 | 0.000479039 |
| CALU | 1.29971378 | 2.24E-08 | 9.94E-08 |
| P3H1 | 1.290396299 | 1.65E-12 | 3.01E-11 |
| TGM2 | 1.270033338 | 0.001328702 | 0.00246759 |
| GREM1 | 1.246702848 | 0.009900851 | 0.01501629 |
| SDC4 | 1.215473169 | 0.00027177 | 0.000568531 |
| PLOD1 | 1.195433359 | 1.44E-08 | 6.73E-08 |
| TNFAIP3 | 1.068832242 | 7.48E-05 | 0.000183999 |
| FN1 | 0.998668964 | 0.001525991 | 0.002801525 |
| LOXL1 | 0.996469397 | 0.000770464 | 0.001460671 |
| COL4A2 | 0.952540328 | 0.000268811 | 0.000568531 |
| COPA | 0.942133065 | 1.44E-08 | 6.73E-08 |
| NID2 | 0.928961701 | 3.40E-05 | 8.85E-05 |
| PCOLCE | 0.922297078 | 0.020055531 | 0.029200854 |
| LAMA1 | 0.820900583 | 0.008524437 | 0.013238385 |
| DAB2 | 0.802832588 | 0.005717522 | 0.009048601 |
| ITGAV | 0.795384154 | 0.000234962 | 0.000509084 |
| ITGB1 | 0.762490998 | 0.000198205 | 0.000445349 |
| WNT5A | 0.762061964 | 1.77E-05 | 4.75E-05 |
| ITGB5 | 0.754291035 | 0.000200981 | 0.00044608 |
| COL16A1 | 0.719496817 | 0.032683931 | 0.04540821 |
| FOXC2 | 0.689612027 | 0.001539299 | 0.002801525 |
| SAT1 | 0.682616785 | 0.035643149 | 0.048606005 |
| VCAM1 | 0.681812459 | 0.008761379 | 0.013399755 |
| ITGA5 | 0.655094406 | 0.0169926 | 0.025349617 |
| GPC1 | 0.626029826 | 0.022275428 | 0.032175619 |
| FBN1 | 0.612367117 | 0.035837398 | 0.048606005 |
| TPM4 | 0.572219336 | 9.72E-05 | 0.000235919 |
| PLOD2 | 0.563103807 | 0.019099171 | 0.028032655 |
| OXTR | 0.51798253 | 7.14E-10 | 5.42E-09 |
| EDIL3 | -0.508511054 | 0.00042487 | 0.000831466 |
| CD59 | -0.523514211 | 0.000337344 | 0.000689849 |
| MAGEE1 | -0.534901632 | 6.33E-05 | 0.000160059 |
| SCG2 | -0.69092835 | 0.000388958 | 0.000769461 |
| SLIT3 | -0.716557384 | 0.001735168 | 0.003096084 |
| PTX3 | -0.745509936 | 2.69E-06 | 7.96E-06 |
| SGCG | -0.832716859 | 9.98E-08 | 4.04E-07 |
| TIMP3 | -0.847753564 | 0.008583129 | 0.013238385 |
| APLP1 | -0.890968928 | 0.00054725 | 0.001048416 |
| SLIT2 | -0.982417621 | 0.002356499 | 0.004008251 |
| FBLN1 | -1.046455061 | 7.98E-06 | 2.20E-05 |
| EMP3 | -1.110848699 | 3.80E-05 | 9.74E-05 |
| PDLIM4 | -1.136067488 | 0.000103887 | 0.000248783 |
| PMP22 | -1.16924902 | 0.001860648 | 0.003287747 |
| FGF2 | -1.253669908 | 0.000382125 | 0.00076425 |
| TGFBR3 | -1.31587157 | 0.000493111 | 0.000954747 |
| CXCL12 | -1.331420714 | 7.52E-07 | 2.58E-06 |
| MFAP5 | -1.398141456 | 7.34E-05 | 0.000182892 |
| JUN | -1.446566956 | 0.002336889 | 0.004008251 |
| FERMT2 | -1.455684596 | 0.02533338 | 0.036020899 |
| GAS1 | -1.482077168 | 0.004783939 | 0.007705105 |
| PCOLCE2 | -1.500193658 | 3.46E-07 | 1.26E-06 |
| DCN | -1.530915995 | 1.64E-06 | 5.34E-06 |
| ACTA2 | -1.677856593 | 0.000245751 | 0.000526197 |
| FLNA | -1.824282214 | 0.036346769 | 0.048640529 |
| RHOB | -1.902467386 | 0.011195893 | 0.016840103 |
| MYLK | -1.939336118 | 0.00037698 | 0.000762337 |
| TAGLN | -2.038413008 | 0.000116182 | 0.000274611 |
| TPM1 | -2.114269518 | 0.003319917 | 0.005543348 |
| TPM2 | -2.300503709 | 0.001042933 | 0.001956844 |
| SFRP1 | -2.33698926 | 6.60E-11 | 6.00E-10 |
| GADD45B | -2.435550811 | 0.000157605 | 0.00036309 |
| ABI3BP | -2.455647166 | 2.18E-06 | 6.72E-06 |
| MYL9 | -2.631731301 | 1.22E-05 | 3.30E-05 |
