# Supplementary material for: Identification of an EMT-Related Gene Signature for Predicting Overall Survival in Gastric Cancer
Source: Front Genet. 2021 Jun 24;12:661306. doi: 10.3389/fgene.2021.661306 (PMC8264558; doi:10.3389/fgene.2021.661306)
Supplement: Supplementary Table 3 — Multivariate Cox regression of TCGA-STAD and GSE62254 cohorts with variables including age, gender, stage, LNR and risk score. [file Table_3.docx]

**Supplementary Table 3.** Multivariate Cox regression of TCGA-STAD and GSE62254 cohorts with variables including age, gender, stage, LNR and risk score.

|  | **TCGA-STAD** | | |  | **GSE62254** | | |
| --- | --- | --- | --- | --- | --- | --- | --- |
| **Characteristics** | **Number** | **Hazard Ratio (95%CI)** | ***p*-value** |  | **Number** | **Hazard Ratio (95%CI)** | ***p*-value** |
| **Age** | 278 | 1.028 (1.006-1.050) | **0.012** |  | 298 | 1.028 (1.012-1.044) | **0.001** |
| **Gender** |  |  |  |  |  |  |  |
| Male/Female | 175/103 | 1.388 (0.878-2.194) | 0.161 |  | 197/101 | 0.996 (0.703-1.411) | 0.982 |
| **Tumor stage** |  |  |  |  |  |  |  |
| II/I | 82/39 | 1.226 (0.552-2.724) | 0.617 |  | 96/30 | 1.679 (0.649-4.343) | 0.285 |
| III/I | 127/39 | 1.248 (0.548-2.845) | 0.598 |  | 95/30 | 2.671 (1.029-6.934) | **0.044** |
| IV/I | 30/39 | 2.946 (1.188-7.307) | **0.020** |  | 77/30 | 4.836 (1.755-13.327) | **0.002** |
| **LNR** | 278 | 3.050 (1.439-6.463) | **0.004** |  | 298 | 7.025 (2.796-17.652) | **<0.001** |
| **Risk score** | 278 | 3.956 (1.943-8.058) | **<0.001** |  | 298 | 4.063 (2.084-7.921) | **<0.001** |
