# Supplementary material for: Identification of an EMT-Related Gene Signature for Predicting Overall Survival in Gastric Cancer
Source: Front Genet. 2021 Jun 24;12:661306. doi: 10.3389/fgene.2021.661306 (PMC8264558; doi:10.3389/fgene.2021.661306)
Supplement: Supplementary Table 4 — Clinical characteristics of TCGA-STAD cohort patients in different risk groups. [file Table_4.docx]

**Supplementary Table 4.** Clinical characteristics of TCGA-STAD cohort patients in different risk groups

| **Characteristics** | **Whole cohort (n=278)** | **Low risk (n=148)** | **High risk (n=130)** | ***p*-value** |
| --- | --- | --- | --- | --- |
| **Age** |  |  |  | 0.100 |
| <60 years | 89 (32.0%) | 41 (27.7%) | 48 (36.9%) |  |
| ≥60 years | 189 (68.0%) | 107 (72.3%) | 82 (63.1%) |  |
| **Gender** |  |  |  | 0.480 |
| Female | 103 (37.1%) | 52 (35.1%) | 51 (39.2%) |  |
| Male | 175 (62.9%) | 96 (64.9%) | 79 (60.8%) |  |
| **Tumor stage** |  |  |  | 0.066 |
| I-II | 121 (43.5%) | 72 (48.6%) | 49 (37.7%) |  |
| III-IV | 157 (56.5%) | 76 (51.4%) | 81 (62.3%) |  |
| **T** |  |  |  | 0.161 |
| T1-2 | 73 (26.3%) | 44 (29.7%) | 29 (22.3%) |  |
| T3-4 | 205 (73.7%) | 104 (70.3%) | 101 (77.7%) |  |
| **N** |  |  |  | **0.021** |
| N0 | 76 (27.3%) | 49 (33.1%) | 27 (20.8%) |  |
| N1-3 | 202 (72.7%) | 99 (66.9%) | 103 (79.2%) |  |
| **M** |  |  |  | 0.207 |
| M0 | 260 (93.5%) | 141 (95.3%) | 119 (91.5%) |  |
| M1 | 18 (6.5%) | 7 (4.7%) | 11 (8.5%) |  |
| **LNR** |  |  |  | **<0.001** |
| low | 228 (82.0%) | 133 (89.9%) | 95 (73.1%) |  |
| high | 50 (18.0%) | 15 (10.1%) | 35 (26.9%) |  |
