# Supplementary material for: Identification of an EMT-Related Gene Signature for Predicting Overall Survival in Gastric Cancer
Source: Front Genet. 2021 Jun 24;12:661306. doi: 10.3389/fgene.2021.661306 (PMC8264558; doi:10.3389/fgene.2021.661306)
Supplement: Supplementary Table 5 — Clinical characteristics of GSE62254 cohort patients in different risk groups. [file Table_5.docx]

**Supplementary Table 5.** Clinical characteristics of GSE62254 cohort patients in different risk groups

| **Characteristics** | **Whole cohort (n=298)** | **Low risk (n=144)** | **High risk (n=154)** | ***p*-value** |
| --- | --- | --- | --- | --- |
| **Age** |  |  |  | 0.206 |
| <60 years | 106 (35.6%) | 46 (31.9%) | 60 (39.0%) |  |
| ≥60 years | 192 (64.4%) | 98 (68.1%) | 94 (61.0%) |  |
| **Gender** |  |  |  | 0.658 |
| Female | 101 (33.9%) | 47 (32.6%) | 54 (35.1%) |  |
| Male | 197 (66.1%) | 97 (67.4%) | 100 (64.9%) |  |
| **Tumor stage** |  |  |  | **0.018** |
| I-II | 126 (42.3%) | 71 (49.3%) | 55 (35.7%) |  |
| III-IV | 172 (57.7%) | 73 (50.7%) | 99 (64.3%) |  |
| **T** |  |  |  | **0.004** |
| T1-2 | 186 (62.4%) | 102 (70.8%) | 84 (54.5%) |  |
| T3-4 | 112 (37.6%) | 42 (29.2%) | 70 (45.5%) |  |
| **N** |  |  |  | 0.569 |
| N0 | 38 (12.8%) | 20 (13.9%) | 18 (11.7%) |  |
| N1-3 | 260 (87.2%) | 124 (86.1%) | 136 (88.3%) |  |
| **M** |  |  |  | **0.042** |
| M0 | 271 (90.9%) | 136 (94.4%) | 135 (87.7%) |  |
| M1 | 27 (9.1%) | 8 (5.6%) | 19 (12.3%) |  |
| **LNR** |  |  |  | **0.003** |
| low | 226 (75.8%) | 120 (83.3%) | 106 (68.8%) |  |
| high | 72 (24.2%) | 24 (16.7%) | 48 (31.2%) |  |
